# Schizophrenia and Bone Marrow Disorders: An Emerging Clinical Association

**DOI:** 10.7759/cureus.75822

**Published:** 2024-12-16

**Authors:** Jayalekshmi Jayakumar, Manasa Ginjupalli, Aju Kalaivani Babu, Srinishant Rajarajan, Madhuri Jakkam Setty

**Affiliations:** 1 Department of Internal Medicine, The Brooklyn Hospital Center, New York, USA; 2 Department of Internal Medicine, Allegheny General Hospital, Pittsburgh, USA; 3 Department of Psychiatry, Texas Tech University Health Sciences Center, Odessa, USA

**Keywords:** bone marrow malignancies, bone marrow transplantation, clozapine, cytokines, immune dysregulation, neuroinflammation, schizophrenia, schizophrenia and hematological malignancies, treatment resistant schizophrenia

## Abstract

Schizophrenia is a chronic psychiatric disorder with a complex etiology involving genetic, neurobiological, and environmental factors. Many individuals with schizophrenia experience treatment resistance despite advances in pharmacologic and non-pharmacologic interventions. Immune dysregulation, characterized by altered cytokine levels, immune-related gene expression, and neuroinflammation, plays a critical role in schizophrenia's pathogenesis. Furthermore, associations between schizophrenia and bone marrow malignancies, with documentation of remission of treatment-resistant schizophrenia following bone marrow transplantation for malignancies, are emerging. This review synthesizes findings on the immunological underpinnings of schizophrenia, explores potential connections with bone marrow dysfunction, and evaluates the therapeutic potential of immune-modulating approaches, including bone marrow transplantation. By highlighting these connections, the study aims to advance understanding and stimulate prospective research into innovative treatments for this debilitating condition.

## Introduction and background

Schizophrenia has been classically defined as a chronic psychiatric disorder influencing early brain development, which has a diverse interplay of genetic and neurobiological factors [1]. It is a burdensome psychiatric disorder affecting approximately 0.25%-0.6% of the US population [2]. The Diagnostic and Statistical Manual of Mental Disorders, Fifth Edition (DSM-5) defines schizophrenia as the presence of two out of five of the following: a combination of psychotic symptoms such as (i) hallucinations, (ii) delusions, (iii) disorganized speech, (iv) grossly disorganized or catatonic behavior and motivational and cognitive dysfunctions, and (v) negative symptoms (affective flattening, alogia, or avolition). Despite the newer development of antipsychotics and non-pharmacologic interventions, treatment-resistant schizophrenia occurs in approximately 30% of individuals diagnosed with schizophrenia [3]. Immune dysfunction and the role of infectious and inflammatory agents in the pathophysiology of schizophrenia were in theory for decades. However, this concept and its implications in management strategies for treatment-resistant schizophrenia never came into the mainstream of research [4]. Emerging evidence suggests an unusual phenomenon - treatment-resistant schizophrenia being cured by bone marrow transplantation in bone marrow malignancies [5]. This raises multiple questions - Is there an association between schizophrenia and bone marrow malignancies? Is there an actual immune element in the pathogenesis of schizophrenia? This study aimed to explore the immune basis of schizophrenia and the role of bone marrow in the pathogenesis and management of treatment-resistant schizophrenia.

## Review

Schizophrenia: Pathogenesis and pharmacologic treatment

While no singular central pathophysiology, diagnostic pathology, or biological marker has been recognized, multiple theories have been proposed, which include both neurodevelopmental and neurodegenerative theories [6]. The neurochemical hypothesis suggests the involvement of neurotransmitters such as dopamine, serotonin, and glutamate in the pathophysiology of schizophrenia [7]. This hypothesis is supported by the confirmed activity of dopaminergic, glutamatergic, and gamma-aminobutyric acid (GABA)ergic systems in patients with schizophrenia [8,9].

The two-hit hypothesis proposes that schizophrenia arises from a combination of genetic predisposition and environmental factors [10]. This notion aligns with the neurodevelopmental hypothesis, which suggests that interactions between multiple susceptibility genes and environmental insults contribute to schizophrenia’s onset [11]. Meta-analyses have demonstrated a high heritability of schizophrenia [11], with linkage studies pinpointing susceptibility loci across various chromosomal regions [12]. Multiple genome-wide association studies (GWAS) have identified variable risk loci, supporting the hypothesis that schizophrenia’s etiology involves numerous genes, each making a small contribution to overall risk [13,14]. The International Schizophrenia Consortium has also endorsed this polygenic model (Table 1) [15].

**Table 1:** Key Pathophysiological Theories of Schizophrenia IL-6: interleukin 6

Theory	Key Description	Supporting Evidence
Neurochemical Hypothesis	Suggests neurotransmitter imbalances, particularly dopamine, serotonin, and glutamate, contribute to schizophrenia.	Dysregulated dopaminergic and glutamatergic activity in the brain.
Neurodevelopmental Hypothesis	Schizophrenia results from abnormal brain development influenced by genetic and environmental factors, often in early brain formation.	High heritability, prenatal exposure to toxins or infections linked to increased risk.
Two-Hit Hypothesis	Combines genetic predisposition with environmental factors, leading to the disorder's onset.	Evidence of genetic loci and environmental insults (e.g., viral infections, stress) influencing schizophrenia onset.
Immune Dysregulation Hypothesis	Suggests immune system dysfunction, including cytokine imbalance, contributes to schizophrenia development.	Elevated IL-6, changes in cytokines, and neuroinflammation are found in schizophrenia patients.

Among these loci, the major histocompatibility locus (MHC) stands out as one of the strongest associated with schizophrenia [13,16,17]. This multifaceted understanding underscores the complexity of schizophrenia’s etiology, with genetic and environmental factors interacting in its development.

Evidence suggests an association between schizophrenia and immune system-related genes such as CD19 and CD20, implying a potential role of immune dysregulation in the disorder [18]. Studies have shown immune dysregulation and alterations in neuroinflammatory pathways in schizophrenia [19,20], possibly linked to dopamine-induced activation of autoimmune T cells [21]. Elevated levels of proinflammatory markers and cytokines [19,20], including interleukin 6 (IL-6), which stimulates B lymphocytes, have been observed in individuals with schizophrenia [19,22]. Nuclear factor kappa B (NF-κB) signaling, natural killer cell signaling, and B cell receptor signaling have also been implicated in the disorder [20]. This cytokine-induced neuroinflammation may lead to microglial activation, contributing to the progression of inflammatory processes and neurodegeneration in schizophrenia [23]. Pharmacological treatments targeting this imbalance in inflammatory cytokines, such as non-steroidal anti-inflammatory drugs (NSAIDs) have shown some efficacy in reducing psychotic symptom severity [20].

**Figure 1:**
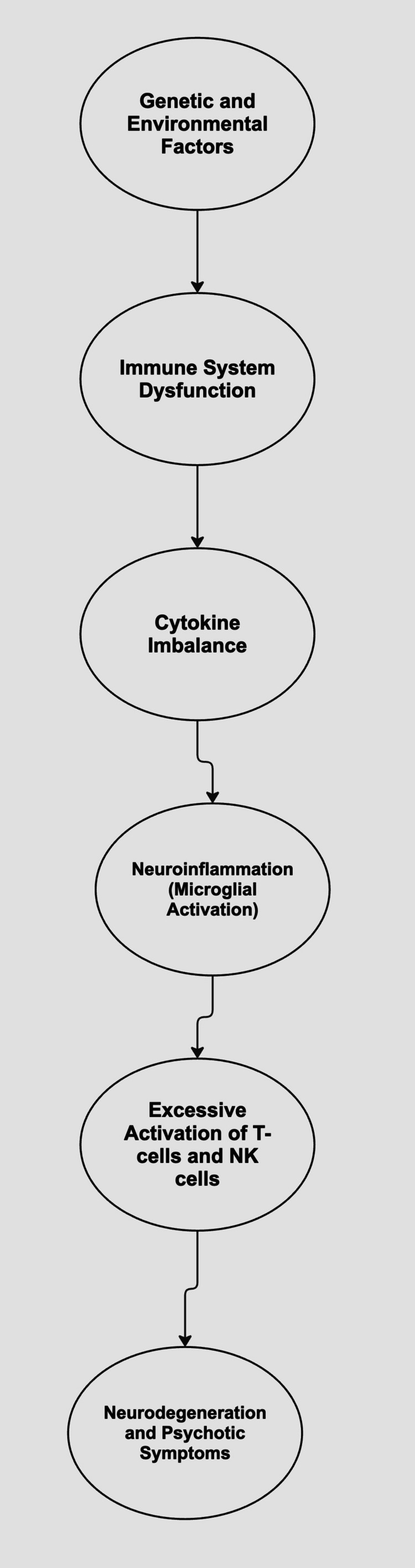
Proposed Mechanism of Schizophrenia Pathogenesis Involving Immune Dysregulation The image was created by J. Jayakumar using Lucidchart.

Moreover, environmental factors, including various childhood and prenatal maternal inflammatory processes, have been implicated in the development of schizophrenia (Figure 1) [24-26]. Despite these insights, the limited understanding of schizophrenia’s pathophysiology hinders the development of successful treatments. Currently, there is no cure and symptomatic treatment is only partially effective [25]. Management primarily revolves around blocking dopamine receptors in the central nervous system. A newer drug - xanomeline/trospium chloride - was recently approved for use in schizophrenia, which targets muscarinic receptors [27]. Monoclonal antibodies targeting inflammatory pathways, coupled with advances in pharmacogenomics and personalized medicine, would optimize antipsychotic efficacy for treatment-resistant cases.

Non-pharmacological treatment options for schizophrenia

Modern approaches include both pharmacological and non-pharmacological approaches to therapy. While pharmacological treatments, including various antipsychotic medications, typically aim at alleviating symptoms of schizophrenia, they often fail to address the underlying causes of the disorder [28]. Moreover, there has been a growing dissatisfaction with the outcomes achieved solely through antipsychotic medication, particularly concerning functional recovery. Coupled with the notable reported rates of medication non-adherence, there has been a notable surge in the development of non-pharmacological interventions for schizophrenia [6]. Hence both pharmacological and non-pharmacological treatments must be used to optimize long-term treatment.

Non-pharmacological treatment options fall into three main domains such as psychotherapy, electroconvulsive therapy, and repetitive transcranial magnetic stimulation, of which psychotherapy remains the most widely used [29].

Individual therapy, particularly cognitive behavior therapy (CBT), focuses on the interplay between cognitive processes, emotions, and behaviors to address symptomatology in mental health disorders like schizophrenia. CBT aims to reduce distress through adaptive cognitive and behavioral strategies, yielding positive outcomes such as symptom reduction and improved social functioning [30-33]. Additionally, mindfulness-based group therapies, including dialectical behavior therapy and acceptance and commitment therapy, have shown effectiveness in managing hallucinations and delusions [34]. Recent holistic approaches, which encompass financial management, independent living skills, and health management, have also gained attention for their positive influence on schizophrenia outcomes [28].

The link between schizophrenia and bone marrow: Evidence from recent studies

It is well-documented that autoimmune diseases and allergies may be transferred by stem cell transplantation, a process mediated by the transfer of donor lymphocytes [35]. A case of schizophrenia transmitted after allogeneic stem cell transplantation from an affected sibling raises the question of such involvement of lymphocytes as a mediator for schizophrenia [36]. This research was revolutionary as it supports the hypothesis of immunological involvement in schizophrenia pathogenesis and suggested to mark this as an opportunity for inducing remission of the psychotic process and as a potential area of research.

Elevated IL-6 and higher neutrophil count from bone marrow hyperactivity are not uncommon in patients with schizophrenia and can be explained as a reflection of immune dysregulation. A novel discovery of elevated neutrophil extracellular traps (NETs) in schizophrenia proposed that this may in fact predispose schizophrenic patients to inflammatory and autoimmune diseases resulting in reduced life expectancy [37]. Studies aimed at this association found NETs and IL-6 to be good predictors and targets in early schizophrenia.

The role of GABAergic neuronal cell grafting in treating schizophrenia has become an emerging area of research recently. To provide an unlimited source of GABAergic cells, bone marrow-derived mesenchymal stem cells were reprogrammed through overexpression of the Achaete-scute homolog 1 (Ascl1, also called Mash1) to generate GABAergic neuron-like cells [38]. This genetic engineering of bone marrow cells as a useful measure to obtain GABAergic neuron-like donor cells for the treatment of schizophrenia bolsters the potential for bone marrow harvest in the treatment of schizophrenia.

Some studies targeted the mechanism of impaired adult hippocampal neurogenesis in the development of schizophrenia and used intracerebroventricular transplantation of bone marrow-derived mesenchymal stem cells to promote hippocampal neurogenesis [39]. Such studies revealed promising results in animal studies; however, its impact on humans is yet to be explored.

Schizophrenia and bone marrow malignancies

Historical bone marrow studies of chronic schizophrenic patients date back to 1964, which showed an increased number of reticulum cells, abnormal lymphocytes, and multinucleated giant cells, which may be related to the abnormal blood proteins seen in schizophrenic patients [40]. However, causation analysis is yet unexplored after six decades. This observation was adopted in recent reviews, which gave valuable insights into the immune basis of schizophrenia [41]. Schizophrenia was shown to be associated with abnormalities in all immune system components: from innate to adaptive immunity and from humoral to cellular immunity. It was also observed that schizophrenia patients had increased evidence of C-reactive protein, dysregulation of cytokines and chemokines, elevated levels of neutrophils and autoantibodies, and microbiota dysregulation [41]. Since the immune system plays a critical role in identifying and eliminating tumors, dysregulation of the immune system is hypothesized to increase the risk of developing cancer [42].

The link between schizophrenia, its management, and bone marrow cancers remains largely uncharted territory in mainstream scientific inquiry, poised for deeper exploration. The most commonly seen bone marrow malignancies are leukemias, lymphomas, and myelomas. Documented instances were largely associated with treatment-resistant schizophrenia [4,5]. Clozapine is the drug of choice for treatment-resistant schizophrenia [43]. It has demonstrated superiority to other antipsychotics and is associated with lower mortality rates despite the risk of agranulocytosis. There's ongoing research into its potential link to hematological malignancies. Some studies over the past 10 years report the risk associated with clozapine in schizophrenia patients in terms of bone marrow cancers, indicating some immunological crossroads between schizophrenia and its treatment with clozapine and the development of bone marrow malignancies [43].

In a study, the incidence of acute myeloid leukemia was higher in schizophrenia patients who received clozapine when compared to those who didn’t receive clozapine (hazard ratio (HR): 8.31, confidence interval (CI): 2.02-34.23) [44]. The exact mechanism of leukemia risk in schizophrenia patients receiving clozapine is unknown, but some theories hypothesize that it may involve cytotoxic effects on bone marrow stroma cells and the creation of nitrenium ions through clozapine oxidation. These ions are believed to act as free radicals, potentially interacting with DNA and leading to an increased likelihood of leukemia [45]. The association between clozapine and an increased risk of lymphoma is also reported [46]. A case of diffuse large B-cell lymphoma was diagnosed in a schizophrenia patient who had been taking clozapine for a long time [47].

A Finland-based case-control and cohort study reported that long-term clozapine use, considered a gold-standard treatment for refractory schizophrenia, is associated with an increased risk of hematological malignancies such as leukemias, lymphomas, and myelomas, compared to other antipsychotics. A complementary analysis showed that risk increase was specific for hematological malignancies, but not for any other malignancies, indicating an underlying immunological phenomenon. Additionally, clozapine use was associated with increased odds of hematological malignancies in a dose-response manner (adjusted odds ratio: 3.35, CI: 2.22-5.05). Long-term clozapine use also had a higher effect on mortality due to lymphoma and leukemia than due to agranulocytosis (26 deaths vs 3 deaths) [48].

Role of bone marrow transplantation in treatment-resistant schizophrenia associated with bone marrow malignancies

While clozapine is undoubtedly studied to be superior to any other drugs in treatment-resistant schizophrenia [43], evidence from large trials revealed mirtazapine, electroconvulsive therapy, and memantine to be the best three augmentation treatments in clozapine-resistant schizophrenia [29]. Newer data suggests that the major underlying pathogenic mechanism of schizophrenia is immune dysregulation. Therefore, a cellular therapy like bone marrow transplantation, which could balance inflammation by immune regulation, has the potential to be effective in patients with treatment-resistant schizophrenia [49].

Even though multiple studies showed a higher incidence of bone marrow malignancies like leukemia in schizophrenic patients, this was explored in detail in a case report where bone marrow transplantation for leukemia led to remission of treatment-resistant psychosis [5]. This serves as supportive evidence for prospective studies exploring this association. The study concludes that bone marrow transplantation definitely possesses the potential to manage overall disease severity and improve the quality of life in treatment-resistant schizophrenia. The study could not exclude the possibilities of spontaneous improvement without any treatment, paradoxical improvement following cessation of neuroleptics, and the curative effect of multiple immune-modulating drugs [5].

## Conclusions

This review underscores the potential link between schizophrenia and bone marrow malignancies like leukemia, emphasizing the role of immune dysregulation in the pathogenesis of the disorder. Historical evidence, coupled with recent findings, supports the hypothesis of an immune origin for schizophrenia and the possibility of therapeutic benefits from interventions like bone marrow transplantation. Given the increasing case reports of treatment-resistant schizophrenia and associated malignancies, further research is crucial to establish causative relationships and refine treatment strategies. By exploring the immunological aspects of schizophrenia, this investigation paves the way for innovative therapies that could significantly enhance patient outcomes and quality of life. Future studies are essential to validate these findings and develop effective management protocols targeting the immune system in schizophrenia.
